# 

**Published:** 1883-04

**Authors:** 


					﻿INTERNATIONAL HAHNEMANNIAN ASSOCIATION.
Bureau of Clinical Medicine : Edward Bayard, M. D., New
York; Constantine Lippe, M. D., New York; C. Carleton Smith,
M. D., Philadelphia; G. Pompili, M. D., Rome, Italy; C. F. Nich-
ols, M. D., Boston; E. A. Ballard, M. D., Chicago; Rollin R.
Gregg, M. D., Chairman, Buffalo.
NOTICE.
All articles for publication, books for review, business communications, etc.,
must be sent to Editor, 2109 Chestnut Street, Philadelphia.
				

